# Achieve Closed Reduction of Irreducible, Unilateral Vertically Displaced Pelvic Ring Disruption with an Unlocking Closed Reduction Technique

**DOI:** 10.1111/os.12958

**Published:** 2021-04-04

**Authors:** Hua Chen, Qun Zhang, Yan Wu, Zuhao Chang, Zhengguo Zhu, Wei Zhang, Peifu Tang

**Affiliations:** ^1^ The Department of Orthopaedic Trauma Chinese PLA General Hospital (301 Hospital) Beijing China

**Keywords:** Closed reduction, Pelvic fracture, Screw fixation

## Abstract

**Objective:**

To be able to treat irreducible unilateral vertically displaced pelvic ring disruption (UVDPRD) using closed reduction, we introduced a technique named Unlocking Closed Reduction Technique (UCRT) and evaluated its effectiveness with improved pelvic closed reduction system (PCRS).

**Methods:**

A retrospective study was performed in our department. Between January 2014 and December 2017, 43 patients whose UVDPRD were not successfully reduced using transcondylar traction. Subsequently, they were treated with UCRT using improved PCRS. The study included 19 male and 24 female patients, with a mean age at the time of the operation of 46.2 years. During surgery, operation time and blood loss were recorded. Post‐surgical reduction quality was evaluated using Matta scoring criteria and patient lower‐extremity functional outcome was evaluated using Majeed functional scoring criteria.

**Results:**

When used with improved PCRS, UCRT achieved pelvic reduction in all 43 cases of irreducible UVDPRD with postoperative pelvic reduction quality rated excellent and good for 42/43 (97.6%) patients according to the Matta scoring criteria (Matta Score < 10 mm). While no post‐surgical complications emerged as the direct result of UCRT in this cohort of patients, 8/37 patients who were treated with subcutaneous supra‐acetabular pedicle screw internal fixation (INFIX) for anterior ring fixation developed lateral femoral cutaneous nerve injury but recovered 6 months postoperatively. No revision surgery was performed on any of the recruited patients. All patients' lower‐extremity functionality was rated excellent with an average Majeed function score of 94.3 during the last follow‐up at an average of 41.6 months postoperatively.

**Conclusion:**

With excellent surgical and functional outcomes in patients with irreducible UVDPRD, improved PCRS‐assisted UCRT proved to be a safe and effective method for the treatment of irreducible UVDPRD.

## Graphical abstract

Unlocking Closed Reduction Technique (UCRT) first unlocks the dislocated iliosacral joint or overlapping edges of the sacral fracture through a force generated by framed‐based unlocking reduction device (FBURD) that pulls the posterior ilium laterally and then corrects the cranial and posterior displacement of posterior ring through transcondylar traction force.

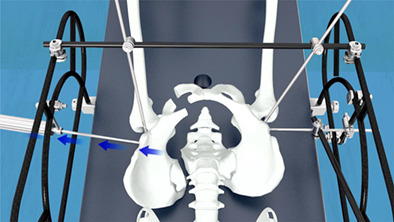

## Introduction

Pelvic ring disruption (PRD) accounts for 3% of all adult fractures, and the majority of these cases, especially cases of irreducible, unilateral vertically displaced pelvic ring disruption (UVDPRD), requires surgical treatment^1^. Although many surgeons prefer open reduction and internal fixation through a posterior approach for achieving an accurate reduction, in fact achieving excellent reductions is so difficult through open reduction in a series of patients with unstable PRD and potential risk of infection still exists due to big exposure^1^, ^2^, ^3^.

Closed reduction through transcondylar traction‐based corridor screw (CS) fixation for adult PRD is preferred over open reduction^4^, ^5^, ^6^, however, this technique also encountered some challenges. A tremendous amount of caudad traction via transcondylar traction is required to reduce the cranially displaced posterior ring, but there are no good methods to resist this transcondylar traction^3^, ^5^, ^6^, ^7^, ^8^. Several tools were developed to resist the transcondylar traction, including Matta frame^9^, Starr frame^10^, ^11^, ^12^, percutaneous Schantz screw in the greater trochanter ^2^, and femoral distractor^4^, ^13^. However, when used with these tools, current closed reduction techniques have difficulties in achieving complete or nearly anatomical reduction of irreducible UVDPRD because hematoma maturation and soft tissue fibrosis often develop, which likely increase the risk of failed closed reduction of irreducible UVDPRD^4^, ^5^, ^8^, ^14^. As a result, open reduction is often employed to treat irreducible UVDPRD, and an accepted closed reduction technique for irreducible UVDPRD is not established.

**Fig. 1:**
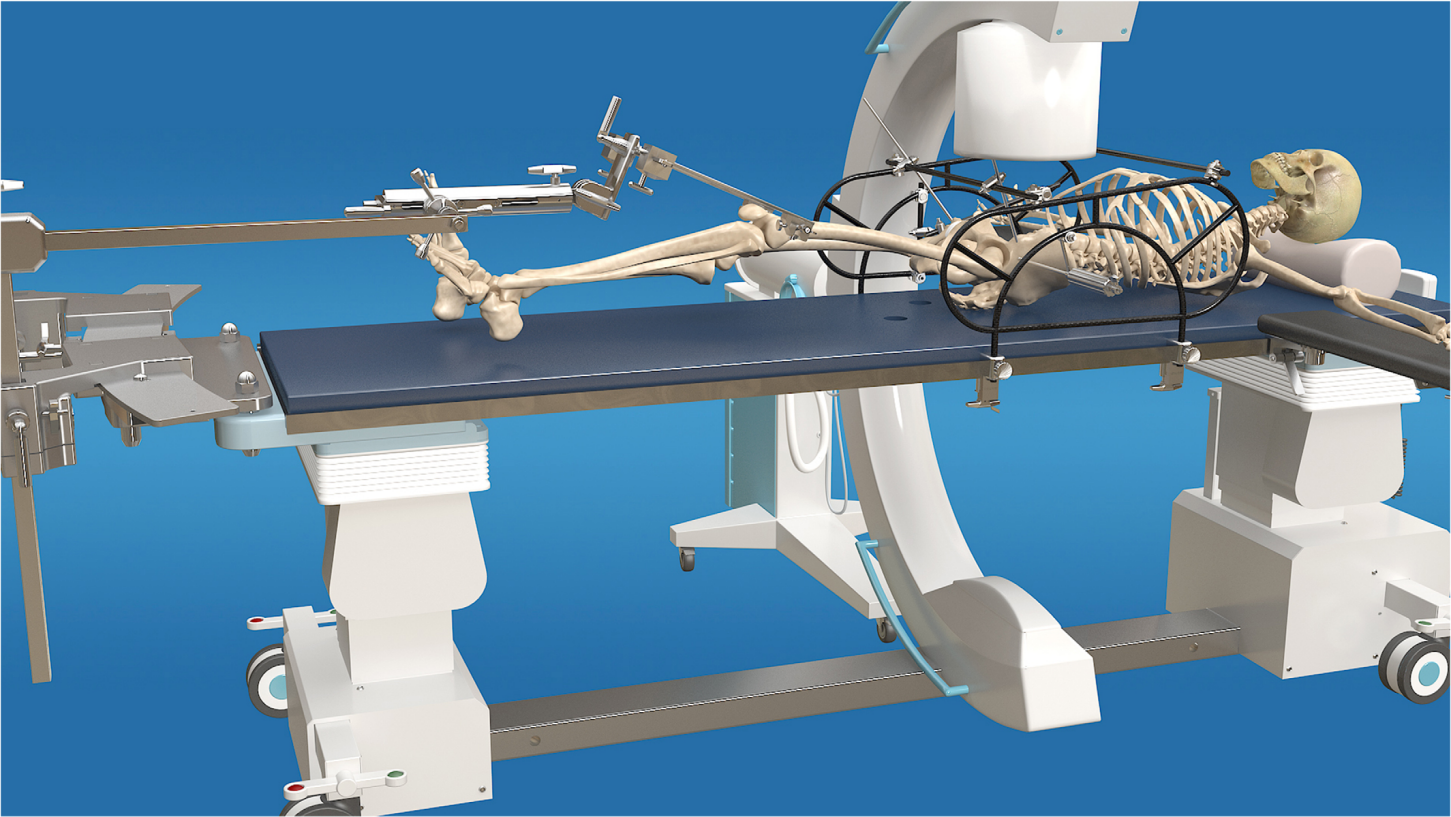
This figure shows the improved pelvic reduction system used in this study.

To be able to treat irreducible UVDPRD using closed reduction, a new technique named Unlocking Closed Reduction Technique (UCRT) is introduced in this study. UCRT's ability to achieve complete or nearly anatomical reduction in patients with UVDPRD is evaluated in this retrospective cohort study using the improved pelvic closed reduction system (PCRS) (Fig. 1), which constitutes the radiolucent surgical table, modified Starr Frame, auxiliary reduction pins, connecting rod and clamp, framed‐based unlocking reduction device (FBURD), and transcondylar traction system. Therefore, the purpose of this study was to assess patients' surgical and lower‐extremity functional outcomes, so as to understand the effects of UCRT during close reduction of UVDPRD.

## Methods

### Study Design

#### General Data

The presented retrospective study was performed with the approval of our institution's human subjects review board. Signed informed consent forms were obtained from all patients enrolled in this study. Patients with UVDPRD who failed to achieve UVDPRD reduction after transcondylar traction between January 2014 and December 2017 were included in our study.

The exclusion criteria were: (i) Age < 18 years old; (ii) Pathological fractures; (iii) No consent for transcondylar traction treatment; (iv) Inability to apply device due to poor local skin conditions; (v) Severe medical complications; and (vi) Other associated severe injuries, such as nerve injury.

**Fig. 2:**
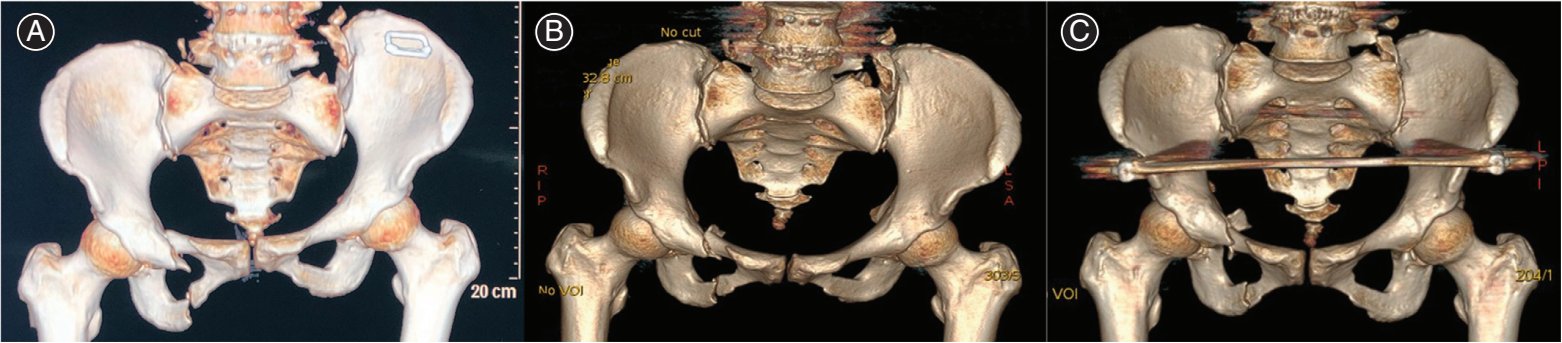
This figure is a computer tomography 3D reconstruction of a recruited patients' initial injury (A), post‐operative reduction quality of transcondylar traction (B), and complete anatomical reduction after receiving unlocking closed reduction technique (C).

**Fig. 3:**
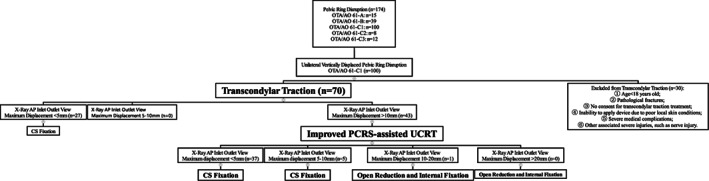
This figure demonstrates the design of this retrospective cohort study. AP, anteroposterior radiograph; Inlet, inlet radiograph (40 degrees caudad); Outlet, outlet radiograph (40 degrees cephalad); CS, corridor screw; PCRS, pelvic close reduction system; UCRT, unlocking closed reduction technique.

A total of 174 patients with PRD were admitted to hospital's level one trauma center, of which 100 patients were diagnosed with UVDPRD (Fig. 2A). Of these 100 patients, 70 patients with normal nerve functions were treated with transcondylar traction immediately after hospitalization. Nevertheless, transcondylar traction failed to achieve UVDPRD reduction in 43 patients as the magnitude of their pelvic fracture displacement after receiving transcondylar traction remained significant (Fig. 2B). These 43 patients were recruited for this study and consented to participate (Fig. 3).

### Improved Pelvic Closed Reduction System (PCRS)

**Fig. 4:**
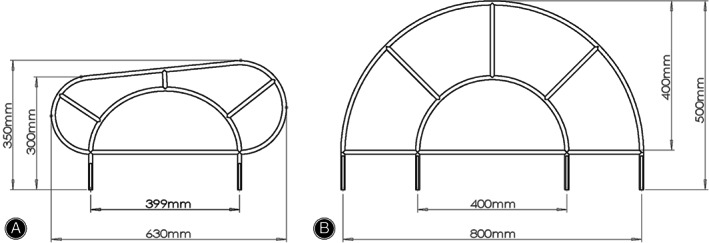
This figure demonstrates and compares the dimensions of the modified Starr frame (A) used in this study and the Starr frame (B).

**Fig. 5:**
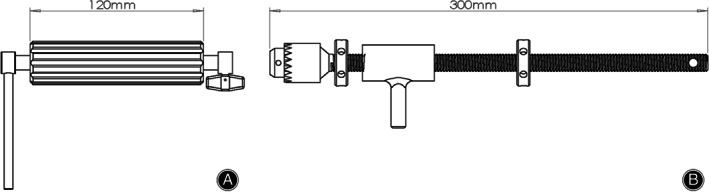
This figure demonstrates and compares the dimensions of FBURD (A) used in this study and the T‐handle (B).

In addition to radiolucent surgical table, auxiliary reduction pins (6 mm in diameter and 40 cm in length), connecting rod and clamp, and transcondylar traction system, the improved PCRS used in this study constitutes two newly developed equipment, modified Starr frame (Fig. 4A) and FBURD (Fig. 5A).

The existing Starr frame has an arch shape (Fig. 4B) and requires a not easily accessible 12‐inch image intensifier for fluoroscopy because the frame is large (length: 0.8 m; width: 0.5 m). By comparison, the modified Starr frame used in this study has an oblique trapezoid shape that better secures the patient during transcondylar traction. Also, the modified Starr frame is significantly smaller (length: 0.6 m; width: 0.3 m) than the existing Starr frame, so fluoroscopy can be achieved through 9‐inch or 6‐inch image intensifier, which are more accessible. Moreover, the modified Starr frame is compatible with intraoperative CT, the iRobot system (TINAVI Medical Technologies Co. Ltd., China) for more accurate CS placement, and all kinds of beds, including wooden beds.

Improving upon the T‐handle **(**Fig. 5B **)**, FBURD is a spin stretcher device that is easier to use, achieving push or pull of the long scans screw through rotation. Also, compared with T‐handle, FBURD has a small size that minimizes interference with fluoroscopy.

### Surgical Process

Step 1: All the patients were performed using general anesthesia. The patient is placed in a supine position on a radiolucent surgical table with a 3‐cm‐thick, padded cushion under patient's lumbosacral region.

**Fig. 6:**
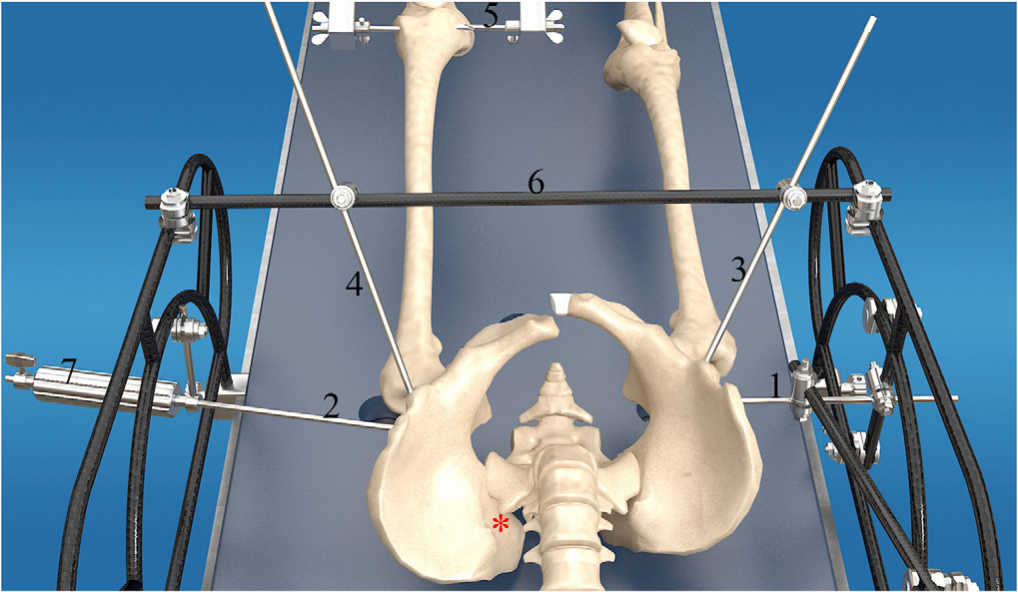
This figure indicates the drilling locations of two transverse supra‐acetabular pins (1, 2), 2 LC‐2 pins (3, 4), and the traction pin (5). This figure also indicates the placement of the connecting rod (6) and FRURD (7), which is connected to the transverse supra‐acetabular pin (2) for unlocking the iliosacral joint dislocation. The red star stands for the dislocated iliosacral joint of the left side of the pelvis.

Step 2: After sterile preparation, PCRS is fixed to the surgical table to build a spatial reduction construct. Reduction pins are symmetrically driven into both sides of the pelvis at the following locations (Fig. 6): (i) two transverse supra‐acetabular pins driven into superior cortex just above the dome of acetabulum under AP view monitoring; and (ii)2 LC‐2 pins driven into pelvis from anterior inferior iliac spine to posterior superior iliac spine under iliac oblique and iliocostal joint up‐down view. For transcondylar traction, the traction pin penetrates femoral condyle and is connected to the traction bow.

Step 3: UCRT is then performed to achieve UVDPRD reduction (OTA tile C1.2 shown with Video S1 and OTA tile C1.3 with Videos S2). The uninjured hemipelvis is secured to the surgical table through the connection of transverse supra‐acetabular pin to the modified Starr Frame and the connection of LC‐2 pin to the connecting rod. Then, the flexion/extension of the injured hemipelvis is adjusted by sliding the connecting rod against the modified Starr Frame. When the connecting rod is horizontally leveled and clamped to the modified Starr Frame, the abduction/adduction of the injured hemipelvis is adjusted by sliding the LC‐2 pin against the connecting rod. The position of the anterior pelvis ring is corrected once the LC‐2 pin on the injured hemipelvis is positioned symmetrically with respect to that of the uninjured hemipelvis and subsequently clamped to the modified Starr frame. Posterior ring reduction is achieved under fluoroscopy. FBURD is connected to the transverse supra‐acetabular pin on the injured side and rotated, pulling the posterior ilium laterally to unlock the dislocated ileocecal joint or overlapping edges of the sacral fracture. Subsequently, transcondylar traction is applied to correct cranial and posterior displacement, setting the posterior pelvis ring in circular motion centered on the connecting point of the LC‐2 pin and the connecting rod. Finally, the transverse supra‐acetabular pin is pushed medially by rotating the FBURD to accurately reduce the posterior ring.

Step 4: Once C‐arm examination reveals satisfactory reduction of the pelvis, an appropriate CS is implanted *via* a minimally invasive approach to fix the fracture. Posterior fixation is achieved through a 7.3 mm, partially threaded trans sacral screw. Anterior fixation is achieved using external fixation or subcutaneous supra‐acetabular pedicle screw internal fixation (INFIX) unless the facture is associated with symphyseal separation, which we treat with cannulated screw fixation before proceeding with anterior fixation.

Once the surgery is completed, the operative time, fracture reduction time, fluoroscopy frequency, and intraoperative blood loss are recorded (Table 1). Also, radiographs of the pelvis were taken to record the reduction quality.

**TABLE 1:** Patients' surgery information

Surgery information	Mean ± SD	Range
Operative time (minutes)	205.7 ± 88.8	70–450
Fracture reduction time (minutes)	100.6 ± 29.4	40–205
Fluoroscopy frequency (times)	146.1 ± 68.9	41–420
Intraoperative blood loss volume (mL)	199.8 ± 297.5	0–1500

Computer tomography 3D reconstructions were performed 3 days postoperatively to determine if all screws were intraosseous. Physical therapy initiated the first day after surgery. On postoperative day one, patients performed isometric quadriceps exercises. On postoperative day two, patients walked with assistance from crutches. Six weeks postoperatively, patients were bearing partial weights. Depending on radiographic assessments of fracture healing, patients were bearing full weights approximately 3 months postoperatively.

Follow‐ups were conducted at 1, 3, and 6 months after the operation and every half year thereafter. At each follow‐up, radiological and clinical evaluations of pelvic fracture were performed, pelvic reduction quality was determined using the Matta radiological scoring system^15^. Patients' functional status were assessed using the Majeed functional scoring criteria at the last follow up^16^.

#### Matta Radiological Scoring

The Matta score is one of the most frequently used tools for the reduction effect of pelvic fracture displacement according to the maximal displacement measured on the anteroposterior (AP), 40° caudad (inlet), and 40° cephalad (outlet) radiographs: Excellent (<=4 mm), good (4–10 mm), fair (10–20 mm), and poor (> 20 mm).

#### Majeed Functional Scoring

The Majeed score is used to evaluate function after major pelvic injuries. Five factors were assessed and scored: pain, standing, sitting, sexual intercourse and work performance. A full score of 100 ∼ 85 was considered excellent, 70 ∼ 84 was good, 55 ∼ 69 was acceptable, and < 54 was poor. The scoring system allows comparison between early and late results and also between various methods of treatment.

### Statistical Analysis

Statistical analysis was conducted using SPSS22.0 software (International Business Machines, corp., Armonk, NY, USA). Paired t‐tests were performed to determine if the difference between the preoperative and postoperative pelvic fracture displacement measurements are statistically significant. Differences for every measurement type are the nearly normally distributed. All data were expressed as mean ± standard deviation (SD). Differences were considered statistically significant when *P* < 0.05.

## Results

### Patients

This cohort of patients includes 19 males and 24 females with an average age of 46.2 years (Range: 18–80 years) and average BMI of 23.2 (Range:18.4–31.1 kg/m^2^). According to the OTA/AO classification, there were 29 cases of type 61‐C1.3 and 14 cases of type 61‐C1.2. According to the Young–Burgess classification, 43 cases are all combined mechanism of injury. 37/43 cases were associated with extrapelvic skeletal injuries. After an average of 10.4 days (Range: 3–34 days) after the injury, patients were treated with UCRT using improved PCRS (Fig. 2C). In addition, all patients were followed up by clinic review. The mean follow‐up period was 41.6 months after the surgery (range, 12–78 months).

### Pelvic Reduction Quality

When used with improved PCRS, UCRT effectively decreased pelvic fracture displacement in the 43 patients enrolled in this retrospective study, demonstrated by the statistically significant differences between preoperative and postoperative displacement measurements on the injured side of the pelvis (*P*‐value<0.01) (Table 2). The postoperative pelvic reduction quality for 42/43 (97.6%) patients were considered excellent and good using the Matta scoring criteria (Matta Score < 10 mm). Specifically, excellent reduction quality (Matta Score < 5 mm) was achieved in 37/43 cases and good reduction quality (Matta Score between 5 and 10 mm) was achieved in 5/43 cases (Fig. 6). Only 1/43 patient's reduction quality was rated fair (Matta Score between 10 and 20 mm).

**TABLE 2:** Preoperative and postoperative pelvic fracture displacement measurements

Measurement type	Preoperative displacement (mm)	Postoperative displacement (mm)	t‐score	*P*‐value
Iliac wing height (AP)	9.6 ± 7.5	1.0 ± 2.5	7.28	0.000
Sacral height (AP)	10.9 ± 8.2	1.3 ± 2.8	7.81	0.000
Ischial height (AP)	9.5 ± 5.9	1.4 ± 2.6	8.33	0.000
Pelvic ring width (inlet)	8.5 ± 8.1	1.3 ± 2.1	5.66	0.000
Sacral width (inlet)	4.6 ± 3.7	0.8 ± 1.6	6.00	0.000
Iliac wing height (outlet)	6.1 ± 8.3	1.4 ± 3.2	3.58	0.001
Ischial height (outlet)	5.8 ± 6.5	1.8 ± 3.2	3.71	0.001

AP, anteroposterior radiograph; Inlet, inlet radiograph (40 degrees caudad); Outlet, outlet radiograph (40 degrees cephalad).

### Complications

Computer tomography 3D reconstructions confirmed that all screws were intraosseous in all 43 patients. No post‐surgical complications emerged as the direct result of UCRT in this cohort of patients. FBURD‐induced neurovascular injury and Schantz pin‐induced ilium fracture—the biggest concerns for UCRT—did not develop in this cohort of patients.

For the 37/43 patients who were treated with INFIX for anterior ring fixation, eight patients developed lateral femoral cutaneous nerve injury—a post‐surgical complication contributable to INFIX—and fully recovered from this complication 6 months after the surgery. INFIX was removed 3 months postoperatively. 6/43 patients were treated with external fixation, which was removed 6 weeks postoperatively. Transsacral and symphyseal screws were left alone. Moreover, no revision surgeries were performed.

### Clinical Improvements

The average fracture‐healing time for the 43 patients is 3.4 months (Range: 3–5 months). At the last follow‐up, all patients were ambulatory with normal gait without any assistive device. Patients' lower‐extremity functionality was measured using Majeed functional scoring system. All patients' functionality was rated excellent with an average Majeed functional score of 94.3 (Range: 80–100). Moreover, all patients returned to their previous employment.

## Discussion

In this retrospective cohort study, the effectiveness of improved PCRS‐assisted UCRT in treating irreducible UVDPRD is investigated using a cohort of 43 patients with pelvic ring disruptions irreducible by transcondylar traction. After receiving improved PCRS‐assisted UCRT, all 43 patients recovered from injury with excellent lower‐extremity functionality.

Transcondylar traction as closed reduction technique for UVDPRD is limited because it fails to achieve complete or nearly anatomical reduction in some cases of UVDPRD. Among the 70 UVDPRD patients who received transcondylar traction initially in this retrospective study, 43/70 (61.4%) patients required further surgery in order to achieve complete or nearly anatomical reduction. Moreover, cases of failed UVDPRD reduction using transcondylar traction are present in previous studies^5^, ^17^. Improved PCRS‐assisted UCRT remedies the limitations of transcondylar traction: Those UVDPRD irreducible by transcondylar traction can be reduced by improved PCRS‐assisted UCRT. Unlike transcondylar traction, which solely corrects the cranial and posterior displacement of posterior ring through transcondylar traction force, improved PCRS‐assisted UCRT first unlocks the dislocated iliosacral joint or overlapping edges of the sacral fracture through a force generated by FBURD that pulls the posterior ilium laterally and then corrects the cranial and posterior displacement of posterior ring through transcondylar traction force. The additional force that pulls the posterior ilium laterally applied in UCRT likely contributes to the procedure's success in achieving pelvis fracture reduction.

Compared to open reduction, closed reduction techniques result in less peeling of the surrounding soft tissues and less secondary damage, which avoids complications that often associate with open reduction ^2^, ^3^, ^4^.No post‐surgical complications emerged as the direct result of UCRT in this cohort of patients, and all patients regained their lower‐extremity functionality indicated by their excellent Majeed functional score.

Nonetheless, this study has its limitations. The effectiveness of improved PCRS‐assisted UCRT in treating irreducible UVDPRD was only demonstrated in 43 patients. However, considering this procedure's success in treating irreducible UVDPRD, further research into the procedure's effectiveness using a randomized, control study with a larger sample size is considered worthwhile. Also, the potential of PCRS‐assisted UCRT in treating bilateral pelvic fractures, a common PRD, is not explored in this study. Considering the similarities between irreducible UVDPRD and bilateral pelvic fracture, the procedure's feasibility in treating bilateral pelvic fractures is worth investigating.

We believe that when used with PCRS, UCRT can become an established protocol for the closed reduction of irreducible UVDPRD because the procedure can effectively achieve what current closed reduction techniques have difficulties with: resolving fracture locking or sticking often involved in cases of irreducible UVDPRD with a force that pulls the posterior ilium laterally ^2^, ^4^, ^5^, ^6^, ^14^, ^15^, ^18^. Especially when fracture locking or sticking aggravates as the patients' wait time for surgery lengthens, PCRS‐assisted UCRT's ability to achieve pelvic reduction in cases of irreducible UVDPRD with lengthy surgery wait time may be particularly useful in developing countries where patients may not immediately receive proper surgical treatment for their pelvic fractures in local hospitals due to lack of infrastructure or doctor training, and the transfer time to a level one trauma center is lengthy.

## Ethical Approval

This study was approved by the ethics committee of the Chinese PLA General hospital (301 Hospital) with approval number 20190831 56321. The study was also registered on ResearchRegistry.com (UNI 5327). This study was performed in accordance with the ethical standards of the Declaration of Helsinki from 1964. All documentation regarding the approval of this study is readily available upon request.

## Supporting information


**Video S1**: UCRT is performed to achieve UVDPRD reduction for OTA tile C1.2.

**Video S2**: UCRT is performed to achieve UVDPRD reduction for OTA tile C1.3.
